# Approach to implant monitoring and data processing with digital implant lifecycle management

**DOI:** 10.1038/s41598-025-99975-w

**Published:** 2025-04-29

**Authors:** Berend Denkena, Marcel Wichmann, Max-Enno Eggers, Crystal Kayaro Emonde, Christof Hurschler

**Affiliations:** 1 Institute of Production Engineering and Machine Tools, Leibniz University Hannover, An der Universität 2, 30823 Garbsen, Germany; 2 Laboratory for Biomechanics and Biomaterials, Department of Orthopaedic Surgery Hanover Medical School, Anna-von-Borries-Strasse 1-7, 30625 Hannover, Germany

**Keywords:** Automatic monitoring, Condition-based maintenance, Database, Digital twin, Implant, Product lifecycle management, TKA, Prognosis, Orthopaedics, Biomedical engineering, Mechanical engineering

## Abstract

Reducing implant failure rates is a primary research objective, involving the development of monitoring methods, new treatment options, improved manufacturing strategies, and innovative implant designs. The goal is to enhance product efficiency across generations by using information from previous iterations to improve patient outcomes. This paper aims to create an information management framework for implant-related data to enhance lifecycle monitoring. Strategies from product lifecycle management, condition-based maintenance, and the digital twin are applied to medical implant monitoring. The proposed digital implant lifecycle management concept records and processes data throughout the implant’s lifecycle, from design and manufacturing to use and disposal. Implemented using an XML-data structure in simulation software, the concept focuses on manufacturing and monitoring, using total knee arthroplasty as an example. A simulation study demonstrates that the digital twin of the implant can simulate various manufacturing scenarios to optimize process parameters, reducing planning efforts for individual implants by up to 28%. The presented concept significantly improves implant and patient monitoring, enhances communication among stakeholders, and allows for scenario simulations to predict implant behaviour and improve future generations.

## Introduction

The failure rates of total knee arthroplasties (TKA) are significantly higher than the failure rates of critical components in aviation, at around 13% within the first 10 years^1^ compared to around 0.0000019%^2^. Continuous health monitoring of components, a proven practice in aviation, represents a promising approach to improving implant longevity and patient outcomes. While past and current research has investigated various methods for assessing implant condition – such as radiographic stereophotogrammetry analysis (RSA) of TKA^3^ – these studies often neglect the subsequent processing, structuring and utilisation of the collected data. However, efficient data management and integration is crucial to making effective use of this information.

To address this gap, this article presents digital implant lifecycle management (DILM) – a structured, lifecycle-oriented approach to managing implant-related data. By integrating Product Lifecycle Management (PLM), Condition-Based Maintenance (CBM) and Digital Twin (DT) technologies, DILM enables continuous monitoring and structured data organisation throughout the entire implant lifecycle. In this paper, implant wear is used as an example of implant status. In the event of critical wear, complications can arise due to wear particles^4,5^, which require a complete revision of the implant. Through the early detection of wear, a partial revision can be performed instead of a complete revision. Furthermore, storing implant-related data in a digital layer improves communication between stakeholders, supports regulatory compliance, and lays the foundation for prediction models that can be used to estimate the lifespan of implants.

This paper aims at the conceptual approach of a data space in implantology using TKA as an example. To increase patient safety through consistent monitoring, a platform is created to manage and exchange all implant-related data. The contribution of this work lies in the conceptualisation and implementation of the DILM framework, which is illustrated using a TKA inlay as a case study. The approach combines concepts from manufacturing technology, such as digital twins^6^, with methods from aviation^7^ and product management^8^. To validate the feasibility and usefulness of this concept, a material removal simulation was integrated into a digital twin environment, demonstrating the potential of structured data management for both production planning and implant monitoring.

### State of the art

#### Product lifecycle management

Product lifecycle management is a business strategy that creates a product-centric environment and ensures the flow of information throughout the organisation and the involvement of all stakeholders throughout the entire lifecycle of a product^9–11^. It uses tools and technologies to provide a common platform for collaboration and streamlining the flow of information in all phases of the product lifecycle^12^. PLM strategies enable the creation, maintenance and storage of product and company information, which makes it possible to find, improve, distribute and reuse data quickly and easily. The aim is to transform the work of employees into a usable and commonly manageable company asset^13^.

In medical technology, these strategies are used to improve communication between the various stakeholders^14,15^. Ngo et al.^16^ showed that the lifecycle of prosthetic implants represents the link between the lifecycle of the disease and that of the prosthesis, and solved semantic problems using a PLM platform. Ardilla-Mejia et al.^17^ applied PLM methods to the development of precision osteosynthesis prostheses, considered the phases of imagination, definition and realisation, and identified the process areas of requirements, design, manufacturing, testing and knowledge management. A phase- and role-specific flowchart should improve the development of PLM software and exchange formats.

#### Condition-based maintenance

Condition-based maintenance focuses on monitoring and maintaining safety-critical components based on their condition, not on a fixed schedule^18^. Suitable parameters are monitored to indicate developing failures, with a focus on error diagnosis and progress^7,19^. To predict errors and estimate the remaining operating time, sufficient data and knowledge of error propagation and failure mechanisms are required^20^. CBM applications are mainly used in areas with many sensors that can be read out, such as manufacturing technology, aviation and rail transport^21,22^.

The advantage is the continuous monitoring and data processing, which is analysed and converted into maintenance recommendations, thus increasing product safety.

These systems are implemented, for example, with the help of the Open System Architecture Condition-Based Maintenance reference architecture^23^. Phillips et al.^7^, for example, derived a hierarchical decision support system that enables the integration of maintenance plans into the fleet operator’s schedule.

#### Digital twins

According to Grieves^24^, a digital twin consists of three components: the physical part, the digital part and the linking mechanism. The physical part represents a real entity such as a product, a machine or a person. The digital part reflects it in real time on the digital layer. The digital twin thus generates knowledge by linking both layers. The linking mechanism enables the bidirectional flow of data between the two parts. The use of digital twins can reduce operating costs and times, increase productivity, support decision-making, improve maintenance plans, enable remote access, increase the safety of the working environment and promote sustainability^25^. One advantage is the efficient transfer and processing of information between the real and virtual levels, which saves human resources.

In healthcare, digital twins are used to optimise operational strategies, capacities, staffing and treatment models. In this context, they are intended to help reduce costs and make informed decisions^26^. One example is the development of a twin by Siemens for the radiology department of a hospital, in which physical entities were transferred to the digital layer and controlled from there, resulting in shorter waiting times, faster patient processing, better equipment utilisation and lower personnel costs^27,28^. In addition to such organisational applications, digital twins are also used in personalised medicine to develop twins for the entire body or a specific organ^29,30^. One example is the ‘HeartModel’ from Phillips, which combines patient data with a generic heart model to create a personalised 3D heart model for the early detection and prediction of cardiovascular diseases^31^.

### Concept of a digital implant lifecycle management

#### Consolidation of the state of the art

**Fig. 1:**
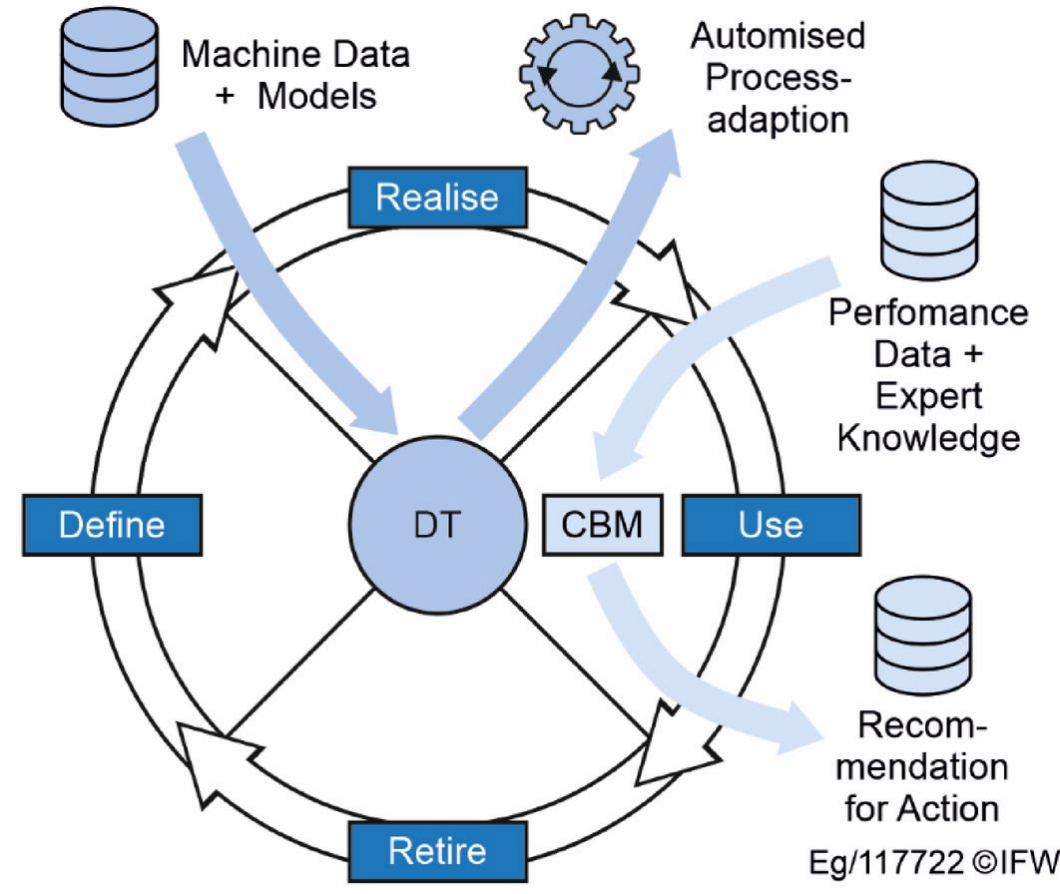
Relationships between the concepts: PLM, CBM and DT.

The concepts presented are correlated by the authors in order to achieve an integrative synthesis the best possible use of each concept’s advantages. It is remarkable that there is a common basis: the centralisation of information and the derivation of activities or recommendations from this data. Nevertheless, different approaches and areas of application are postulated. Product Lifecycle Management, for example, stands for the communication and data exchange of generic products in a corporate environment and works as part of a data platform. Condition-based maintenance is limited to use in the use phase and independently bundles information into recommendations for action in order to minimise the maintenance effort for certain technical components. The digital twin combines both approaches and creates a bidirectional transfer of information into and out of the system. For example, quality deviations in production can be automatically detected and the process parameters can be adjusted by the digital twin. Figure 1 shows an example of the relationship between the three concepts.

The introduced concepts are used to monitor and optimise the functional mechanism and to improve communication between the stakeholders involved in the implant. The aim is to use the totality of data for a single, specific implant from different perspectives. The focus is on the patient, medical staff and the manufacturer/developer.

The basis for the development of a new strategy concept is product lifecycle management, in which information is clearly structured, stored and made accessible. The CBM concept is used to process sensor data or information from measurement procedures (e.g. X-ray images) and to generate recommendations for action for medical personnel. The concept of the digital twin is initially used in manufacturing, where applications already exist^4^.

#### Conceptualisation of digital implant lifecycle management

**Fig. 2:**
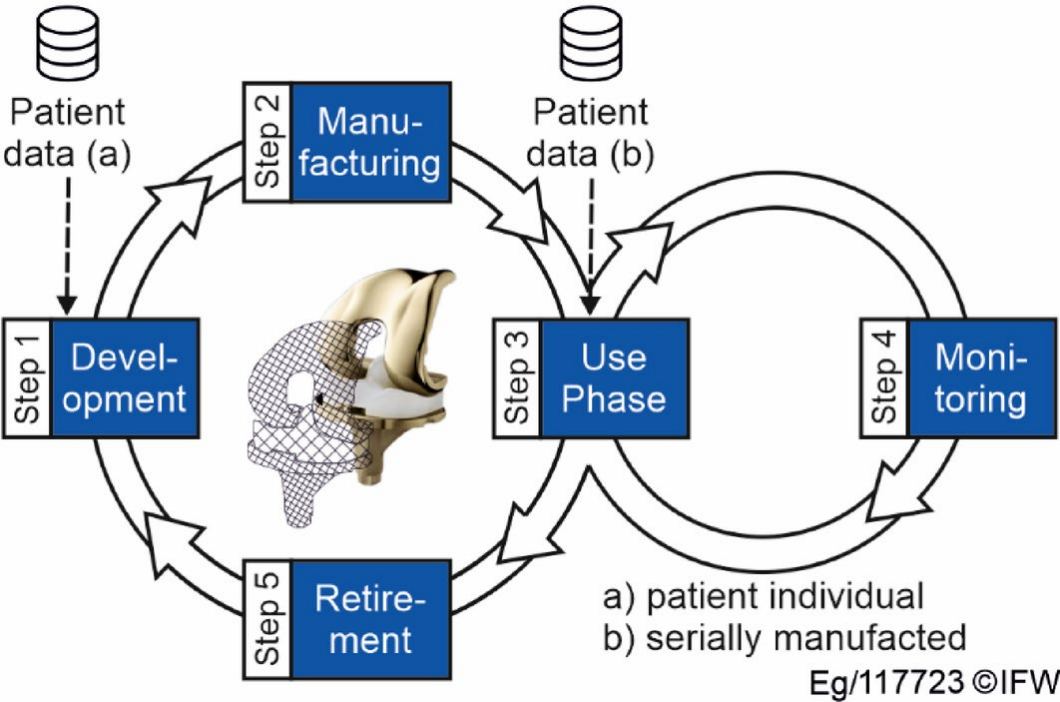
Lifecycle phases of DILM.

In order to design a system that can map the lifecycle of implants, the basic layer is structured on the foundation of a PLM system. To this purpose, a concept was designed that is technically based on both the ‘classic’ product lifecycle management according to Stark^32^ and the definition of the digital twin according to Grieves^24^. This concept is called Digital Implant Lifecycle Management (DILM) and represents the different phases that a specific implant goes through during its lifecycle, as shown in (Fig. 2). When patient data is incorporated into the implant lifecycle, a distinction must be made between patient-specific implants (a) and serially manufactured, non-personalised implants (b), since the former are primarily responsible for the shape of the implant.

The first phase is the development of the implant, which mainly involves design and planning/modelling. If a patient-specific implant is required, a connection to the patient is established in this phase and patient-specific information and data are implemented. In this phase, the DILM primarily serves as a communication tool for the development team and thus fulfils the role of a PLM system. In the second stage, the DILM takes on the characteristics of a digital twin during manufacturing. Various models can be integrated to enable manufacturing simulations that allow the configuration of process adjustments or online process monitoring and thus automatic adjustment of process parameters. The third phase describes the use phase. In this step, the patient data is linked to the implant for the first time if it is a serially produced implant. This means that the patient data is stored in the DILM. This can include both general information (name, address, medical history, etc.) and information specific to the operation or implant (medical history report, operation report, discharge report). Mechanical systems permanently and automatically retrieve information to monitor the state of health. This process is not possible for implants, due to external circumstances (power supply, embedding of sensors in the tissue, etc.). Therefore, a fourth step, the monitoring, was included, which deals with information and data from control examinations by medical specialists or doctors. The data from the implant monitoring are processed and made available in the system so that the responsible body can process them further. The fifth step, the decommissioning, begins with the explantation of the implant and the collection of the associated data. If necessary, additional information is also collected.

A comparison with similar lifecycle concepts in the literature shows that most of these approaches primarily deal with design and manufacturing, but not with monitoring during the use phase^16,17,33^. Mostly the literature deals with approaches coming from product lifecycle management. Systems based on the methods of digital twins and condition-based maintenance could be suitable for a change in condition during monitoring due to their reactive nature. Only Ngo et al.^16^ consider the prosthesis during the use phase, but neither data is collected nor conclusions are drawn from the use phase to other lifecycle phases.

In contrast to the DILM, the patient and not the implant is at the focus of general perception. From the perspective of the healthcare system, the patient must therefore be included in the conceptualisation of the DILM. In this example, the patient can be abstracted from the disease which leads to treatment with an implant. One of the challenges in designing a digital implant twin is the integration of the associated disease lifecycle. This lifecycle describes the different phases that the patient goes through until recovery: health problem, treatment, recovery and end of treatment.

**Fig. 3:**
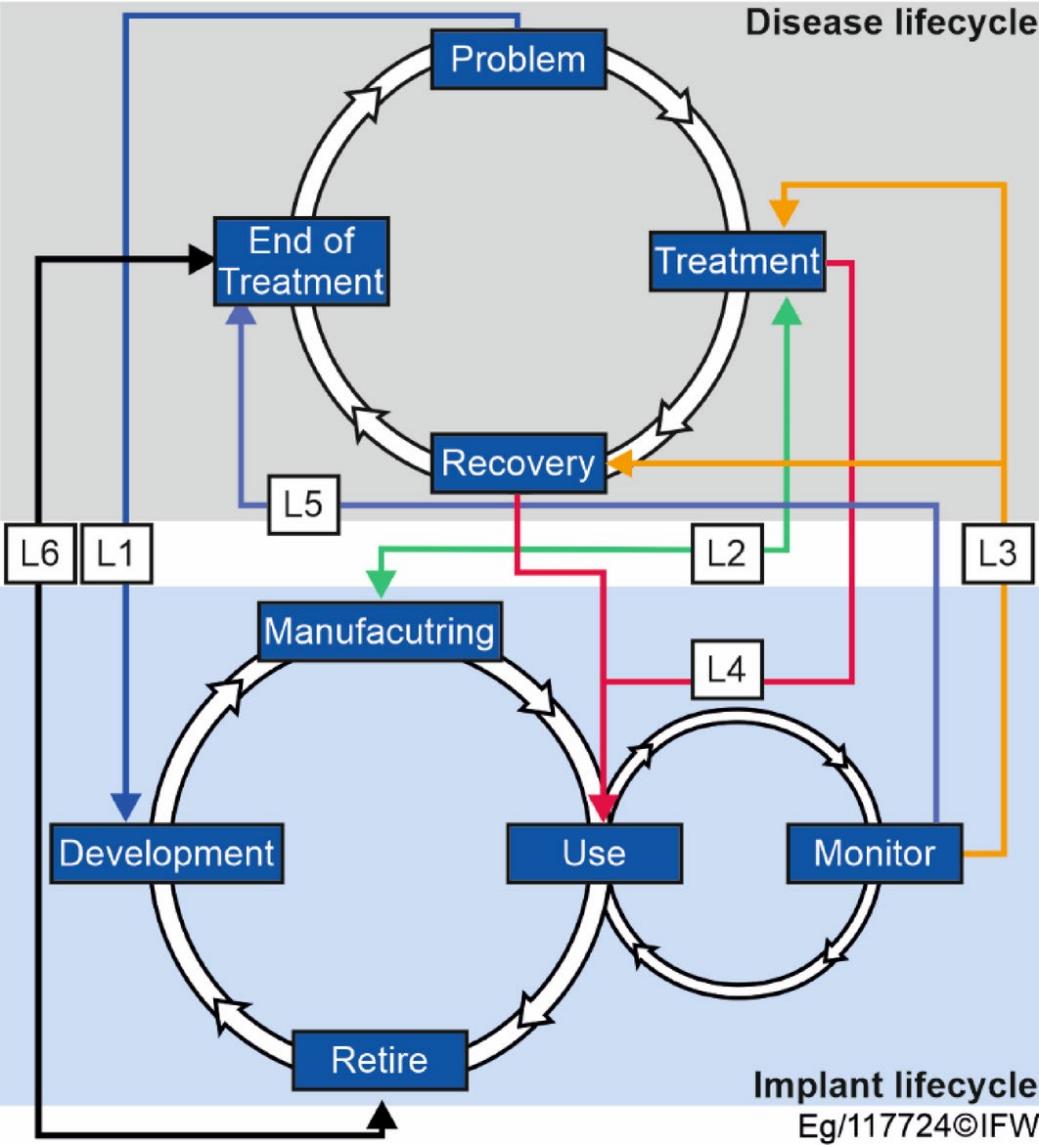
Different links between the disease lifecycle and implant lifecycle.

The links (L1-L6) in Fig. 3 represent the exchange of data or the influence of certain actions. L1 describes the transfer of information from the healthcare professionals to the manufacturer. The manufacturer adapts the implant design to the patient’s condition and constitution. Link L2 shows the interaction between the manufacturer and the healthcare professionals during and after manufacturing. The implant is checked by hospital staff during implantation. On the other hand, the manufacturer sets a delivery date, which allows the operation to be scheduled. Links L3 and L4 represent the information that is accessed during treatment and recovery in the use phase. Link L3 represents information from the monitoring, which is used to further adapt the conventional treatment. Link L4 represents information from the adapted therapy that influences the implant during the use phase. At the same time, appropriate monitoring can lead to the end of treatment if the implant is not functioning correctly (L5). The end of treatment marks the end of the implant’s lifecycle (L6), so they influence each other.

These connections lead to a complex workflow in the implant’s lifecycle and are therefore included in the DILM. The actors involved must also be considered in the structuring of the DILM. The central entity is the patient who receives the implant. The implant, whether customised or serial, is adapted to the patient’s needs. The physical conditions are essential for a successful implantation and the retention of the implant. The producer determines the design, the material and the basic mechanism. The producer can be divided into the design team, which develops the concept and determines the geometry, the material and the surface properties, and the manufacturing team, which produces and checks the required specifications. The producer is not necessarily responsible for both teams, as a contract manufacturer can also be responsible for the production process.

Another entity are the medical professionals. They are primarily responsible for the implantation as well as the pre- and post-operative care. The need for the implantation, the selection of a suitable implant and the follow-up (usually with imaging) are organised and performed around the patient. It can be divided between the hospital staff, who confirm the necessity and are centrally responsible for the operation, and the medical specialist, who makes the initial indication and is responsible for follow-up and regular health and function checks. The manufacturer’s sales department and any research institutions that examine the implant after explantation are also involved, but will not be considered further in this work.

### Realization of concept

**Fig. 4:**
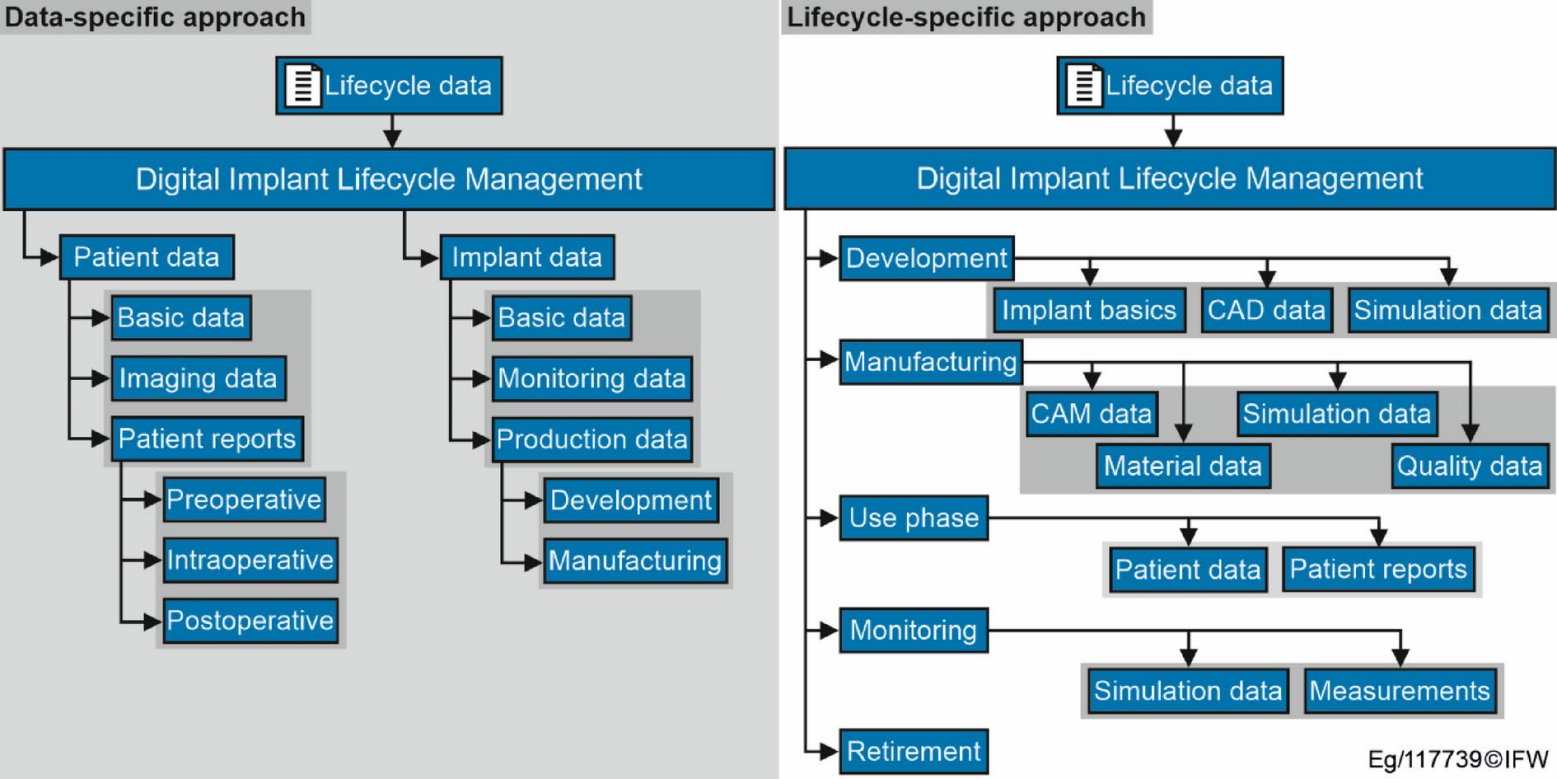
Data-specific (left) and lifecycle-specific (right) structural approaches for the implant data management.

Different approaches are examined for the realisation of a concept. Two different hierarchical structure approaches are considered: a data-specific approach (Fig. 4, left) and a life-cycle-specific approach (Fig. 4, right).

The data-specific approach differentiates between patient-related and implant-related data. Patient-related data is divided into basic information such as age, weight, BMI and imaging data, which is usually collected preoperatively or shortly after surgery. In addition, patient reports are divided into preoperative, intraoperative and postoperative. The implant-related data is divided into basic data such as manufacturer and product number, as well as surgical instructions, monitoring data, i.e. information obtained during the use phase (X-ray images, biofilm data, etc.) and production data. This includes development data (CAD models, surface determination, drawings) and manufacturing data (CAM data, quality control, simulation data).

The lifecycle-specific approach (Fig.4, right) is based on the phases during the lifetime of the implant, as shown in (Fig. 2). Therefore, the data generated in each phase is further subdivided. In the development phase, the data is divided into CAD-related data (3D models, drawings, other geometric information, etc.), basic information (product information, surgical instructions, etc.) and simulation data used to optimise the functional mechanism of the implant. In the manufacturing phase, the data is divided into the following subcategories: CAM data (NC code, process control variables, process monitoring data, etc.), quality data (data collected during quality control), material data (information on the material used in accordance with the Supply Chain Act) and simulation data, which is collected either before or during the process and is used to optimise or monitor it. The use phase is characterised primarily by the hospital stay. It is divided into basic patient information and patient reports, which are based on the data-specific structure approach. The monitoring category is divided into monitoring data (obtained using various measurement methods) and simulation data (uses measured and other data to predict the condition of the implant). The last main category is the retirement and associated data such as explantation and disposal.

Both approaches have different advantages. The benefit of the data-specific approach is an intuitive classification of the different stakeholders to the corresponding information. Accordingly, healthcare professionals can find the data relevant to them by following the categories ‘patient data’. As the DILM is intended to be a communication medium, this is an area of great importance. However, a clear assignment of the monitoring data is not possible in this approach, as it relates to both the patient and the implant itself. It is also possible to assign X-ray images to both ‘imaging data’ and ‘monitoring data’. The lifecycle-specific approach offers a more objective assignment option here. The duplication of information that is stored is significantly reduced. A combination of both approaches is also possible. The life cycle-specific approach can be extended with metadata from the data-specific approach in order to increase the findability of the data based on the metadata and to include both concepts. However, this possibility will not be considered further in this paper for the time being. Based on existing PLM and DT strategies^34^, the concept is implemented in the next chapter using the example of total knee arthroplasty and a lifecycle-specific approach is chosen. Nevertheless, the data-specific approach is an interesting alternative and will be further considered in future research.

With the system structure presented, it is possible to map the entire lifecycle of an individual implant. All information and files can be assigned to exactly one class and made accessible to the responsible entities.

### Implementation using TKA-Inlay

**Fig. 5:**
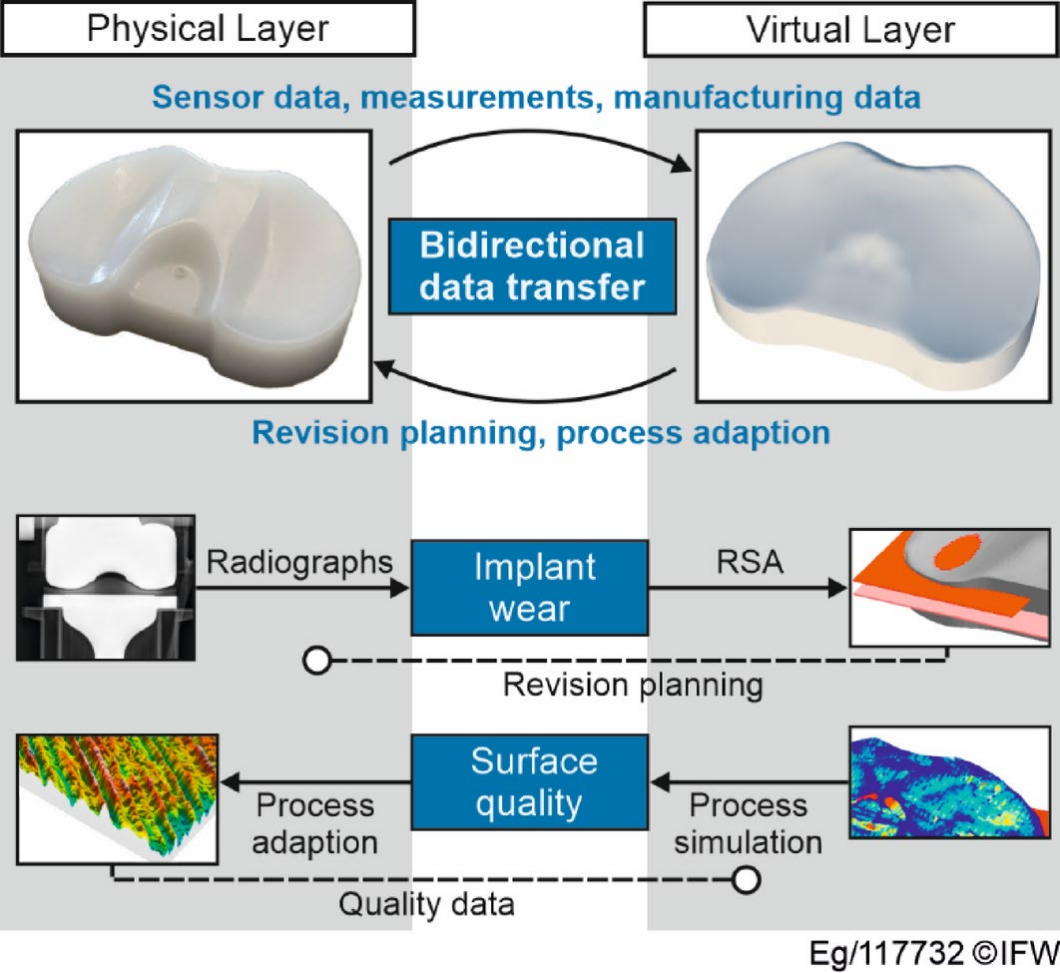
Digital implant twin: Connections between physical and virtual layer.

The TKA is used in this work for the implementation of the DILM. The TKA usually consists of three parts: the femoral component, the tibial component and the implant inlay. The latter is in most cases made of a polymer and together with the metallic femoral component forms a tribological system. Therefore, the inlay is subject to the associated wear phenomena. Excessive wear can lead to revision of the entire implant. In addition to wear-related issues, implant failure can also result from osteolysis, a process where wear particles trigger an inflammatory response, leading to bone resorption and implant loosening. Furthermore, mechanical failure between components, such as delamination, fractures, or loss of fixation, may compromise implant stability. These failure mechanisms must be detected early to enable timely intervention and extend the implant’s lifespan. To address these challenges, this work focuses on monitoring implant wear using the RSA method, which allows the determination of the minimum joint space width (mJSW) as an indicator of inlay degradation over time. Although no exact position in the transverse plane of the implant can be found, the mJSW can serve as a measure of the condition of the inlay. This method has already been investigated^35,36^, and the data obtained from it is used in this work as an example of monitoring data. The visualisation of a specific implant in the virtual layer and the connection of the digital implant twin is shown in (Fig. 5). Information from the real world, such as the implant ID or material, can be digitally stored and analysed in this way.

In the first step, a data set with information from the different lifecycle phases was collected. The data is representative and cannot be assigned to a specific product or patient. The data set used is listed in Table 1 and consists, for example, of CAD data, various documents, production data and X-ray images. The available patient reports were structured according to the model of real reports and then completed using AI tools to avoid the use of real patient information. The X-ray images used are images of a phantom knee, a mechanical device that simulates the position of the implant in the human body. The X-ray images were also stored in DICOM and JPEG format to obtain a diverse data set.

While Table 1 primarily lists file types, it is essential to consider the specific data points handled within these files. These include geometric CAD data of the implant, material properties, manufacturing parameters such as tool paths and cutting forces, clinical monitoring values like mJSW measurements, and diagnostic information from imaging techniques. By explicitly listing these data points, we ensure that critical information is systematically structured to support effective analysis and decision-making throughout the implant lifecycle. The hierarchical XML format ensures clarity and traceability, as it organizes data according to the lifecycle-specific structure outlined in (Fig. 4). Different nodes within this structure store data, files, and metadata, making it possible to flexibly integrate and retrieve various types of information. This approach allows for efficient management of complex datasets in compliance with regulatory requirements.

**Table 1:** Used data for the implementation.

Data title	Data type	Size [KB]
CAD File of the inlay	.stl	223
Manufacturer’s Surgical Instructions	.pdf	6021
Information on material incompatibilities	.pdf	291
NC-code for milling	.mpf	1877
Raw part geometry	.stl	1
Data sheet for raw part material	.pdf	576
Simulated process data	.dat	47,214
Process data recorded during manufacturing	.csv	10,046
Patient’s admission report	.pdf	95
Surgical report	.pdf	132
Discharge report	.pdf	326
Radiograph (pre-surgical)	.dcm	3564
Radiograph (post-surgical)	.jpg	46
Wear data from RSA	.csv	3
In total	–	70,425

The cutting simulation IFW CutS is used for the application of the DILM^37^. This software for kinematic-geometric process simulations maps a tool/workpiece interaction model. IFW CutS has already been used to model digital workpiece twins by capturing axis positions and machine data in parallel to the process in order to analyse the cutting conditions, using external models to adjust the process parameters^38,39^. In this case, data from production can be captured, stored and, if necessary, adjusted in the DT. This structured recording ensures that all relevant information is systematically organised throughout the entire life cycle of the implant. This not only enables effective analysis and decision-making, but also seamless traceability of development, production and usage data. The data is stored and exchanged in a hierarchically structured XML format that ensures clarity and traceability. The structure is based on the tree diagram in Fig. 4 (right), in which different nodes are provided for data, files and metadata. This structure ensures that both production-relevant information – such as CAD models, manufacturing parameters and material properties – and clinical monitoring data, such as mJSW measurement values, can be stored and retrieved in a targeted manner. A key advantage of this flexible XML format is the ability to efficiently integrate different data sources from the various life cycle phases of an implant. This ensures consistent and standardised management of complex data sets that meets regulatory requirements. In addition, the format facilitates the exchange of data between the stakeholders involved – from manufacturers to hospitals and research institutions – and enables long-term data analysis to optimise implant designs and production processes.

The CAD file, the manufacturer’s operating instructions and information on material incompatibilities are assigned to the first phase (development). In this case, the hierarchical structuring of the data is carried out via data and file nodes, which can be arranged in any number of levels. Various viewers are included for visualising different file types. For example, CAD models, images or text files can be displayed.

**Fig. 6:**
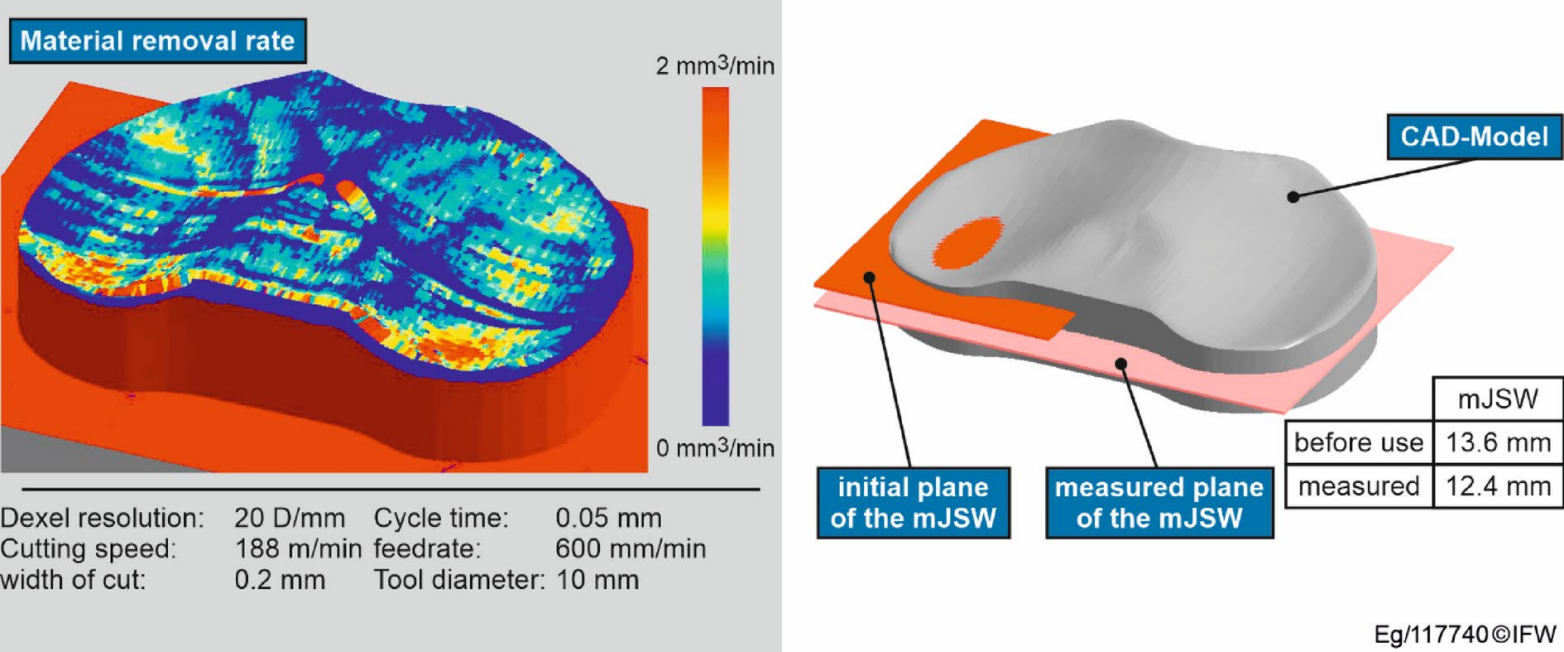
Left: Simulation of implant manufacturing with the DT of the inlay. Right: Visualization of the measured mJSW compared to an unworn inlay.

The second phase (production) stores information about the raw part (geometry and datasheet), process data (simulated and recorded) and the NC code. The option of production simulation (in parallel with the process or before it) means that kinematic parameters and process parameters, such as the local material removal rate, can be read out and displayed (see Fig. 6, left). As already described, this phase also offers the option of adapting production models, which can be used to correct and improve the process. By continuously recording and monitoring the process, quality control in accordance with the medical device regulation (MDR) can be included.

Furthermore, the simulation can also be used to reduce the planning effort in the manufacturing phase. This is demonstrated in this paper using a simulation study that also focuses on the production of an inlay for a TKA using a 5-axis milling machine. For an optimal manufacturing result, it is assumed that an average material removal rate of 0.29 cm3/min is required to achieve a certain surface roughness. For the CAM planning, three different values for the feed rate (500 mm/min, 600 mm/min and 700 mm/min) were calculated that could lead to the desired result. In conventional production, the product is manufactured at all three feed rates and the surface is then analysed. The calculated production times are 1.03 h, 0.92 h and 0.83 h, with the measurements to determine the surface roughness taking 0.75 h each. 39 min are required to simulate a scenario. The corresponding scenario is then created and evaluated. The corresponding calculations are shown in (Table 2). Conventional production planning requires a total of 5.03 h of planning effort, while simulation-based production planning requires 3.62 h. This results in a time saving of 28%.

**Table 2:** Calculation of the planning effort.

Conventional production			Simulation-supported		
Qty	Title	Time (h)	Qty	Title	Time (h)
1	Manufacturing A	1.03	3	Simulation	0.65
1	Manufacturing B	0.92	1	Manufacturing	0.92
1	Manufacturing C	0.83	1	Analysis	0.75
3	Analysis	0.75			
	In total	5.03		In total	3.62

In the third and fourth phases (use phase and monitoring), the sample data set is created from the patient reports and X-ray images, as well as the measurement results of the RSA. The DILM serves as a central data storage that can be used by the responsible employees for cross-departmental collaboration. This enables different specialists to exchange data more easily and to visualise diagnostic results easily. In this paper, these results are represented by the measurement of the mJSW. The measurement results are stored as a CSV file and visualised as a layer, as shown in Fig. 6 on the right. This layer is located at the height of the measured mJSW in relation to the bottom of the implant inlay. By comparing the mJSW of an inlay that has not been used with the mJSW of an inlay that has been used, it is possible to conclude about the wear of the implanted inlay.

The data stored in the DILM can be read out by the involved stakeholders for further data processing. This can be done by means of reverse transformation from the XML format or the IFW CutS software. In addition, the program creates a folder in which all the files used are stored in unmodified form. For the example of the TKA, the entire lifecycle of the product can be monitored. In particular, functions are already available for production and monitoring during the use phase. In the case of implant monitoring, it is also possible to store additional models in order to simulate various wear scenarios.

## Discussion

The application of the described strategies, product lifecycle management, condition-based maintenance and digital twins, to the field of orthopaedics opens up innovative ways to monitor and extend the lifetime of knee endoprostheses. These approaches provide a systematic and forward-looking view of the implant lifecycle, thereby enabling improved patient safety and more efficient use of resources.

PLM provides a comprehensive platform for capturing, structuring and managing all relevant information throughout the entire lifecycle of a product. In the context of TKA, such a platform enables improved data exchange and communication between the parties involved, such as medical staff, manufacturers and patients. CBM, on the other hand, focuses on monitoring and diagnosing the condition of safety-critical components. In implantology, this can be realised by integrating sensor data or imaging techniques such as X-ray stereo photogrammetry analysis. DT combines both approaches by means of bidirectional data exchange between the physical and digital worlds, thus opening up a wide range of possibilities for process optimisation and early fault diagnosis. While traditional PLM/MES/CRM systems offer comprehensive frameworks for managing standard manufacturing processes, they often lack the flexibility and detailed tracking necessary for medical devices like implants. Medical products are subject to stringent regulatory requirements, such as the Medical Device Regulation (MDR), which mandates a high level of traceability throughout the entire lifecycle. Standard PLM tools, even after configuration, typically cannot handle the level of detail and specific data structures required for medical implants, especially in terms of long-term performance monitoring and compliance with safety standards. In particular, the continuous tracking of implants from production through their use phase requires specialized tools that can manage and integrate various data types, such as CAD models, process parameters, patient-specific data, and in-use performance. This is where DILM and digital twin technologies provide significant advantages, offering a dynamic and detailed approach to managing and optimizing implant performance over time.

The presented Digital Implant Lifecycle Management concept shows what an integrated approach to managing the lifecycle of implants can look like. By using a lifecycle-specific structural approach, a clear and intuitive organisation of the data is made possible, which does justice to the different phases and actors. The successful implementation and simulation of the DILM using the example of a TKA inlay demonstrates the practical applicability and the potential advantages of such systems. The use of real and synthetic data illustrates the flexibility of the system, which can be used for both production planning and monitoring during the use phase. In particular, the integration of mJSW measurement into the Digital Implant Lifecycle Management (DILM) framework enables a seamless connection between production data and long-term implant monitoring. The simulation-based production planning approach demonstrated in this study reduces planning time by 28% while optimizing process parameters such as feed rate and material removal rate. By structuring and storing relevant manufacturing data, such as CAD models, process parameters, and NC code, within a standardized XML-based framework, the system ensures traceability and facilitates compliance with MDR requirements. Furthermore, the ability to store and visualize patient-specific wear data based on mJSW measurements allows for a continuous feedback loop between production and clinical outcomes, ultimately contributing to improved implant longevity and patient safety.

## Conclusions and outlook

This paper shows that the integration of advanced methods from aviation and manufacturing technology into implantology offers promising ways to improve implant lifetime and patient safety. The concept of Digital Implant Lifecycle Management provides a sound basis for effectively monitoring and controlling the various phases of the implant lifecycle.

The implementation of a DILM system offers several advantages:

- Enables detailed and continuous monitoring of the implant.
- Improves communication between the involved stakeholders.
- Supports data-driven decision-making.
- The application of the lifecycle-specific approach shows a clear structuring of the data and thus facilitates collaboration and data exchange.

Future research could aim to further expand the functionalities of the DILM system. This could include the integration of additional monitoring and diagnostic tools, as well as the analysis and optimisation of the collected data through machine learning. In addition, the concept of the digital twin offers further interesting approaches for personalised medicine, e.g. by developing patient-specific simulation models based on an even more detailed data basis. In addition, an extension of the concept to various other implants in the form of a reference architecture, similar to the reference architecture model industry 4.0 (RAMI 4.0)^34^, is being considered.

The long-term vision is to contribute to increasing the lifespan and reliability of implants by continuously improving and adapting DILM technologies, while at the same time making the work of medical professionals easier and ultimately increasing patient safety and satisfaction.

## Data Availability

Data analyzed during the current study are available from the corresponding author upon reasonable request.
